# Cholesterol Laundry of Cell Membrane and Fatty Liver by Detergent Liposomes to Improve Anti‐Cancer Drug Responsiveness of Patient Liver Tissues

**DOI:** 10.1002/advs.76144

**Published:** 2026-06-17

**Authors:** Chansik Kim, Joo Kyung Noh, Sewoom Baek, Seung Eun Yu, Jueun Kim, Seongyo Lee, Youngji Oh, Dai Hoon Han, Seyong Chung, Hak‐Joon Sung

**Affiliations:** ^1^ Department of Medical Engineering Graduate School of Medical Science Brain Korea 21 Project Yonsei University College of Medicine Seoul South Korea; ^2^ Department of Medical Engineering Yonsei University College of Medicine Seoul South Korea; ^3^ Department of Integrative Biotechnology Yonsei University International Campus Incheon South Korea; ^4^ Department of Surgery Division of Hepato‐biliary and Pancreatic Surgery Severance Hospital Yonsei University College of Medicine Seoul South Korea; ^5^ Division of Cardiology Department of Internal Medicine Severance Cardiovascular Hospital Yonsei University College of Medicine Seoul South Korea

**Keywords:** cell membrane, cholesterol laundry, liposome, liver therapy, patient tissue

## Abstract

Cholesterol accumulation in the plasma membrane hinders cellular drug uptake, which often limits therapeutic effects on hepatocellular carcinoma (HCC). This study aimed to develop a liposomal strategy based on its proven clinical biosafety, well‐known membrane trafficking behavior, and potential to act as membrane cholesterol‐specific detergents. Addition of cholesterol or detergent (Triton X‐100) into liposomal membranes laundered cell membrane cholesterol equivalently, thereby improving cellular uptake of drug molecules. However, only the detergent‐containing liposomes caused cytotoxicity as liposomal concentrations increased, justifying the use of cholesterol (+)‐liposomes. The hypoxic status of patient HCCs elevated lipidation, which was laundered by liposomal treatment. Consequent drug uptake and therapeutic effects were improved in cancer cells, 3D spheroids, and patient‐derived HCC tissues using perfusion‐based 3D chip systems. Uptake of hydrophobic drug was also improved to a greater extent compared to hydrophilic ones across these models. In mouse fatty livers, IV injection of cholesterol (+)‐liposomes alone decreased hepatic lipidation, inflammation, and fibrosis. When the plasma membrane forms endosomes by encapsulating the liposomes after uptake, membrane cholesterol is transferred from the endosomes to the liposomes and subsequently metabolized in the endoplasmic reticulum (ER). This mechanism was validated using endosome‐mimetic nanovesicles and biochemical ER regulators. The results indicate the promising potential of cholesterol (+)‐liposomes to improve HCC therapy.

## Graphical abstract

Cholesterol‐enriched plasma membranes in hepatocellular carcinoma impede drug penetration. Cholesterol (+)‐liposomes act as membrane‐specific detergents, extracting cholesterol and reducing barrier function without cytotoxicity. Following endocytosis, cholesterol transfers from endosomes to liposomes and is metabolized in the ER. This enhances drug uptake and therapeutic efficacy across cells, spheroids, patient tissues, and fatty liver models.

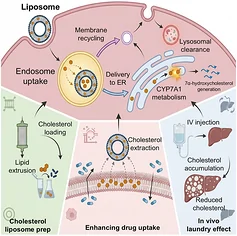

## 1 Introduction

The plasma membrane protects intracellular organs as a versatile barrier of molecular transport [1]. The amphiphilic bilayer of the membrane is constructed from phospholipids, with hydrophilic phosphate groups displayed outward and hydrophobic lipids integrated between the layers, which establishes the barrier function [2, 3]. The hydrophilic phosphate layer facilitates molecular access from the water‐rich environment, while the hydrophobic lipid layer controls molecular passage by repelling water and anchoring transmembrane proteins [4]. Regardless of the transport mechanisms, including passive diffusion, phagocytosis, and endocytosis, trans‐membrane penetration of molecules or drugs is commonly required to enable transport. Consequently, cholesterol deposits in the plasma membrane significantly hinder penetration of molecules and drugs; transport of hydrophobic ones in particular can be further impaired due to hydrophobic interactions [5, 6]. Cholesterol‐rich lipid rafts reinforce this barrier property against drug penetration by reducing membrane flexibility and permeability, and signal transduction is also disrupted as the organization of membrane signaling proteins becomes altered [2]. Therefore, cholesterol accumulation not only physically restricts trans‐membrane transport of molecules and drugs but also alters membrane signaling. In this regard, cholesterol laundry of the plasma membrane represents a potential strategy to improve cellular drug uptake, pointing to a clinical need for membrane cholesterol‐specific detergents.

Liposomes offer specific advantages as a solution, beyond the well‐established fact that their clinical safety has been demonstrated in real‐world applications, and their capacity to deliver drugs into target cells has been studied extensively worldwide [7]. From a mechanistic standpoint, liposomes are produced by the same process that constructs the plasma membrane, yet provide a versatile template for modifying membrane composition, such as through the addition of cholesterol or detergent. As liposomes penetrate from outside the cell, they draw the cholesterol‐rich plasma membrane inward, causing it to form endosomes with cholesterol‐rich membranes by encapsulating liposomes [8, 9]. In this way, cholesterol is depleted from the plasma membrane, and this laundry process continues through cholesterol transfer from the endosome to the liposomes. The endosome then undergoes fission, whereby one part carries the liposomes to the endoplasmic reticulum (ER) for digestion and excretion, while the liposome‐free part is recycled back into the plasma membrane [10]. These two processes are analogous to the washing and drying steps of laundry, respectively.

HCC is a major global health burden and a leading cause of cancer‐related mortality worldwide [11, 12]. The primary risk factors for HCC include hepatitis virus infection, alcohol consumption, and non‐alcoholic fatty liver disease, all of which drive malignant transformation of hepatic cells through chronic inflammation and fibrosis [13]. Although therapeutic modalities for HCC have continued to advance, treatment efficacy remains limited in advanced stages due to persistent resistance and poor responsiveness to anti‐HCC therapeutics [14]. As a test organ for liposomal laundry, the liver is the primary site of cholesterol synthesis and clearance in the body [15]. Lipid accumulation is a major driver of fatty liver formation, which progresses through multiple stages, from inflammation to fibrosis and ultimately to hepatocellular carcinoma (HCC), as membrane lipid is utilized as a cellular energy source [16, 17]. When HCC develops under normal oxygen supply (normoxia) with sufficient vascularization, cellular uptake of anti‐cancer drugs is effectively maintained, aided by low cholesterol levels in the plasma membrane that facilitate drug transport [18]. In contrast, HCC under low oxygen conditions (hypoxia) is drug‐resistant with poor prognosis, and membrane cholesterol levels rise substantially, impeding drug penetration and thereby increasing resistance [19]. This metabolic reprogramming involves incremental cholesterol synthesis and decremental cholesterol excretion in response to cellular stress. In the ER, CYP7A1 converts cholesterol into a relatively more water‐soluble form (7ɑ‐hydroxy), which is subsequently metabolized and effluxed into bile for clearance, which is disordered in hypoxia [20].

Furthermore, IV injections of most therapeutics, including liposomes, accumulate in the liver from circulation before being cleared, making targeted delivery strategies unnecessary [21]. This study therefore, utilizes liposomes with cholesterol incorporated into the membrane, which exerts equivalent laundry effects to the insertion of detergent (Triton X‐100). As a representative outcome, drug uptake by cells, spheroids, and tissues from HCC patients is improved by liposomal pre‐treatment. Because cholesterol preferentially interacts with hydrophobic drugs to impede their penetration, the cholesterol laundry works more effectively in uptake of hydrophobic molecules compared to hydrophilic ones. As the hypoxic status of HCC liver increases, membrane levels of cholesterol are elevated, and thus, the laundry effect and consequent drug uptake are enhanced accordingly. Additionally, IV injection of the liposomes alone significantly reduces fatty liver status, inflammation, and fibrosis, suggesting promising potential for HCC prevention. The role of the ER with a CYP7A1 in cholesterol metabolism following endosomal transport of liposomes is validated using an activator and inhibitor of a key transcription factor (Rev‐Erb ɑ). Together, this study aims to determine (i) whether cholesterol (+)‐liposomes can function as a detergent tool to launder membrane cholesterol; (ii) whether this laundry function can improve drug uptake and therapeutic responsiveness in HCC patient tissues; and (iii) whether membrane laundry itself can be applied to treat mouse fatty liver. We further investigate (iv) whether this effect becomes more pronounced under hypoxic conditions with elevated lipid accumulation in the HCC membrane, and (v) whether intracellular cholesterol trafficking from the endosome to the ER contributes to the underlying mechanism.

## 2 Results

### 2.1 Detergent Effect of Liposomes on Cholesterol Laundry to Improve Cellular Drug Uptake

**FIGURE 1:**
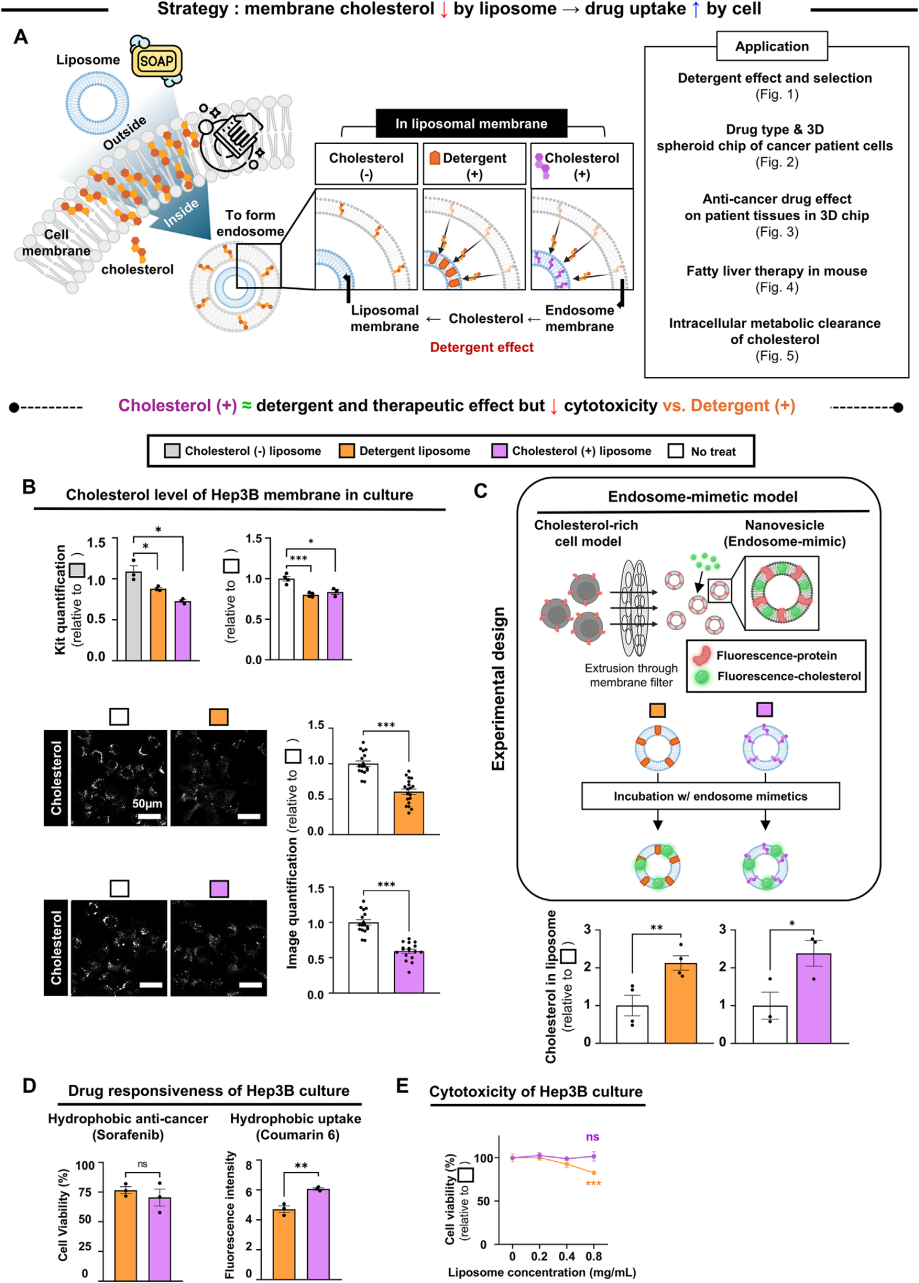
Detergent effect of liposomes as a laundry key of membrane cholesterol to improve cellular drug uptake. (A) Detergent effect of liposome is strategized to launder cholesterol of plasma membrane so that cellular drug uptake is improved. As key mechanistic processes, liposomes penetrate the cholesterol‐rich plasma membrane which directly forms the endosomal membrane with the encapsulation of the liposomes. In this way, cholesterol is transferred from the plasma membrane to the endosomal membrane and finally to the liposomal membrane, which is defined as the soap‐like detergent mechanism. Because cholesterol aggregates with cholesterol through hydrophobic interactions, cholesterol is inserted into the liposomal membrane to form cholesterol (+)‐liposomes with the expectation to improve the laundry function. Moreover, as a real detergent component, Triton X‐100 is added to form Detergent (+)‐liposomes, and these two laundry‐specific liposomes are compared in addition to Cholesterol (−)‐liposomes or No treat for a series of applications (right box). (B) After culturing the hepatocellular carcinoma (HCC) cell line (Hep3B) for 24 h, treatment of either cholesterol (+)‐ or detergent (+)‐liposomes significantly reduces the cholesterol level of plasma membrane compared to cholesterol (−)‐liposomes (top left) and No treat (top right) by kit analysis. These results are validated by image analyses (middle and bottom), indicating the detergent‐like effect of cholesterol (+)‐liposomes. (C) An endosome model is produced by first increasing the cholesterol level of membrane through Hep3B culture in hypoxia, followed by serial filter extrusion to produce NVs. When the NVs are produced, green fluorescence (BODIPY)‐cholesterol is added to the self‐assembly process of membrane so that the detergent effect to transfer green cholesterol from the endosome NV to liposomes can be tracked. Moreover, CD133 of endosomal membrane is labeled with a red fluorescent to distinguish it from the test liposomes. When endosome NVs and test liposomes are incubated for 24 h, and nanoFACS analysis is carried out, green cholesterol is transferred from red endosome NVs to cholesterol (+)‐liposomes significantly more compared to No treat, which is equivalent to treatment of Detergent (+)‐liposomes. (D) After Hep3B cells are treated with test liposomes, subsequent 24‐h treatment of hydrophobic Sorafenib exerts non‐significant difference in the ant‐cancer effect between Cholesterol (+)‐ and Detergent (+)‐liposomes as shown by cell viability. In contrast, cholesterol (+)‐liposomes more effectively facilitates uptake of hydrophobic Coumarin‐6 with fluorescence label from subsequent 6‐h treatment compared to Detergent (+)‐liposomes by imaging analysis. The result indicates a superiority of cholesterol in addition to liposomal membrane to the detergent one. (E) This result is supported by the lower Hep3B cytotoxicity of cholesterol (+)‐liposome compared to Detergent ones when the liposomal concentrations are increased up to 0.8 mg/mL, thereby justifying the use of cholesterol (+)‐liposomes for the follow‐up experiments. Data = mean ± SEM. N = the number of dots as independent replicates in each graph. Statistical significance is denoted by ^*^ *p* < 0.033, ^**^ *p* < 0.002, ^***^ *p* < 0.001, and not significant (ns).

The detergent effect of liposomes is strategized to launder plasma membrane cholesterol so that cellular drug uptake is improved (Figure 1A). As key mechanistic processes, liposomes penetrate the cholesterol‐rich plasma membrane, which directly forms the endosomal membrane by encapsulating the liposomes. In this way, cholesterol is transferred from the plasma membrane to the endosomal membrane and finally to the liposomal membrane, which is defined as the soap‐like detergent mechanism. Because cholesterol aggregates with cholesterol through hydrophobic interactions, cholesterol is inserted into the liposomal membrane to form cholesterol (+)‐liposomes, with the expectation of improving the laundry function. Moreover, Triton X‐100 is added as a real detergent component to form Detergent (+)‐liposomes, and these two laundry‐specific liposomes are compared alongside Cholesterol (−)‐liposomes or No treat for a series of applications (right box).

As Triton X‐100 concentration in the liposomal membrane increases over 0.13 mm, the circular morphology of the liposome is disrupted under transmission electron microscopy (TEM). (Figure S1A). Cell viability also decreases below 80% when the Triton X‐100 concentration exceeds 0.13 mm, with validation of Triton X signals in the liposomal membrane (Figure S1B). Based on these findings, 0.13 mm of Triton X‐100 was selected for incorporation into the liposomal membrane. The average size of the resulting detergent (+)‐liposomes is 103 nm as determined by nanoparticle tracking analysis (NTA) (Figure S1C). Cholesterol (−)‐liposomes also exhibit circular morphology and a diameter of approximately 100 nm under TEM, confirmed by NTA (avg. 98 nm) (Figure S2A).

After culturing hepatocellular carcinoma (HCC) cell line (Hep3B) for 24 h, treatment with either cholesterol (+)‐ or detergent (+)‐liposomes significantly reduces the plasma membrane cholesterol level compared to cholesterol (−)‐liposomes and No treat, as assessed by kit analysis (Figure 1B). These results are validated by image analyses, confirming the detergent‐like effect of cholesterol (+)‐liposomes. Treatment with cholesterol (−)‐liposomes and No treatment results in no significant difference in cholesterol level (Figure S2B). An endosome model is produced by first increasing membrane cholesterol levels through Hep3B culture in hypoxia, followed by serial filter extrusion to produce NVs (Figure 1C). During NV production, green fluorescent (BODIPY)‐cholesterol is incorporated into the membrane self‐assembly process, allowing the detergent effect to transfer green cholesterol from the endosome NVs to liposomes can be tracked. Additionally, CD133 of the endosomal membrane is labeled with a red fluorescent marker to distinguish it from test liposomes. When endosome NVs and test liposomes are incubated for 24 h, and nanoFACS analysis is carried out, green cholesterol is transferred from the red endosome NVs to cholesterol (+)‐liposomes significantly more compared to No treat, at a level equivalent to that of Detergent (+)‐liposomes.

After liposomal pre‐treatment, hydrophilic doxorubicin is applied to Hep3B cells by increasing the concentration for 24 h, and cell viability is assessed by CCK‐8 assay (Figure S2C). Only the highest concentration (10 µm) of doxorubicin significantly reduces cell viability following pre‐treatment with cholesterol (‐)‐liposomes compared to No treat. In contrast, cholesterol (+)‐liposomes support the anti‐cancer effect of hydrophilic doxorubicin across 0.1, 1, to 10 µm as the cell viability significantly decreases compared to No treat. In the setting, the treatment of hydrophobic Sorafenib exerts a non‐significant difference in anti‐cancer effect between Cholesterol (+)‐ and Detergent (+)‐liposomes as shown by cell viability (Figure 1D). In contrast, cholesterol (+)‐liposomes more effectively facilitate the uptake of hydrophobic Coumarin‐6 with fluorescent labeling over a subsequent 6‐h treatment compared to Detergent (+)‐liposomes, as assessed by imaging analysis. This result indicates the superiority of cholesterol addition to the liposomal membrane over the detergent one. This finding is further supported by lo wer Hep3B cytotoxicity of cholesterol (+)‐liposomes compared to Detergent ones as the liposomal concentrations are increased up to 0.8 mg/mL (Figure 1E), thereby justifying the use of cholesterol (+)‐liposomes in follow‐up experiments. Furthermore, a hemolysis assay reveals that cholesterol (+)‐liposomes at 0.2–0.8 mg/mL do not induce hemolysis of red blood cells across varying osmolarities adjusted by NaCl concentration, with no concentration‐dependent difference observed even at 0.55% NaCl, where hemolysis is highly sensitive (Figure S2D). In addition, cholesterol (+)‐liposomes show no cytotoxicity toward healthy endothelial cells (HUVEC) (Figure S2E).

### 2.2 Superior Uptake of Hydrophobic to Hydrophilic Molecules Post Cholesterol Laundry

**FIGURE 2:**
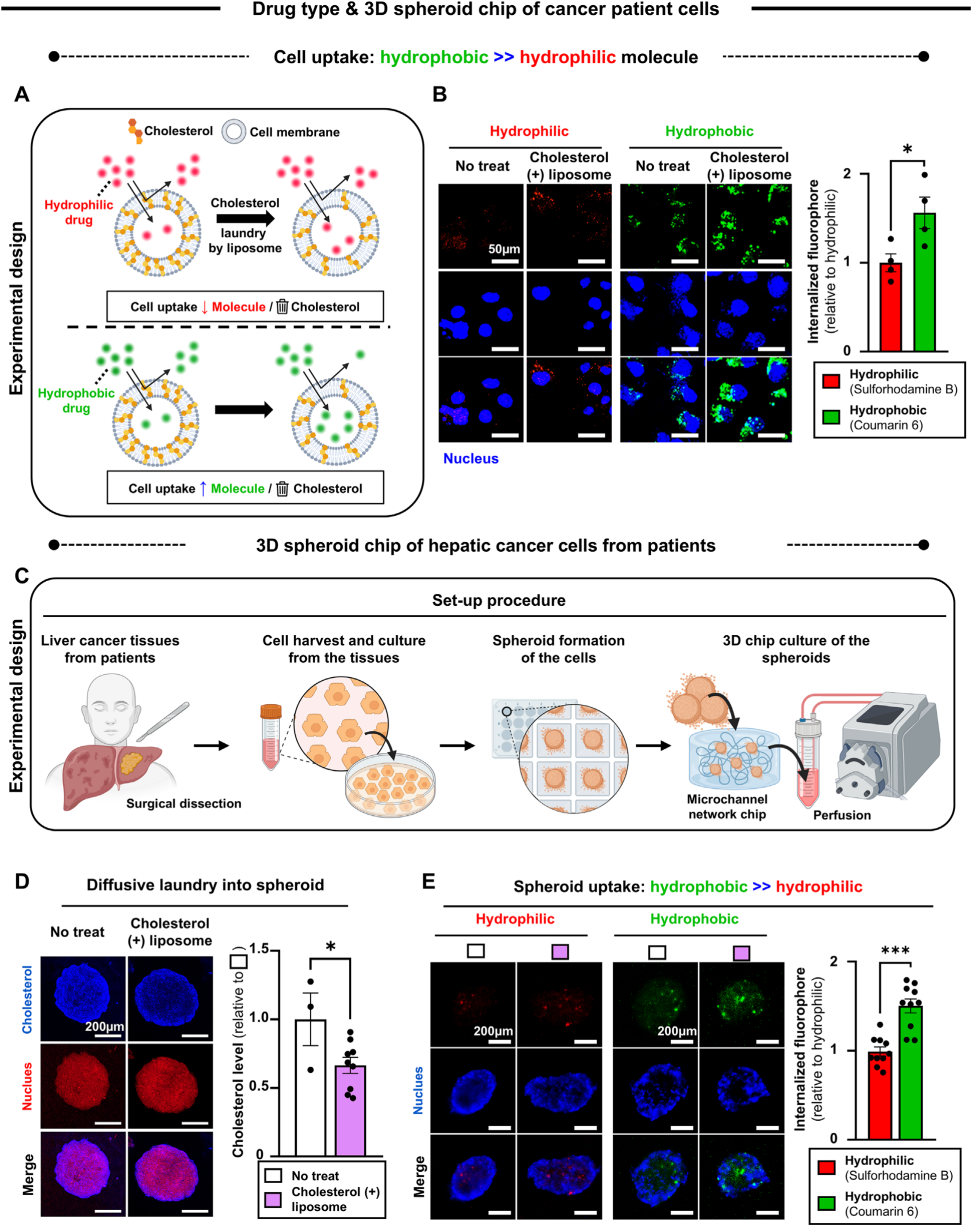
Superior uptake of hydrophobic to hydrophilic molecules post laundry of membrane cholesterol by cholesterol (+)‐liposomes (A) The membrane cholesterol hinders the penetration of hydrophobic molecules through lipidic aggregation. Hence, the laundry of membrane cholesterol by cholesterol (+)‐liposomes is expected to facilitate uptake of hydrophobic molecules more effectively (bottom) rather than hydrophilic ones (top). (B) As validation, Hep3B uptakes hydrophobic Coumarin‐6 (green) significantly more than hydrophilic Sulforhodamine B (red) for 4 h after laundry by cholesterol (+)‐liposomes for 24 h. (C) In addition to the cell culture model, a lump model of HCC is produced by first harvesting cancer tissues from patients upon surgical dissection with isolation and primary culture of patient cancer cells from the tissues, followed by spheroid formation in AggreWell plates. These spheroids are loaded and cultured in 3D microchannel network chips under continuous perfusion without (No treat) or with treatment of cholesterol (+)‐liposomes. (D) As the cholesterol level (blue) is determined under imaging with the identification of nucleus (red), cholesterol (+) liposomes significantly reduced the level compared to No treat, validating exertion of the diffusive laundry effect into the spheroids. (E) The 24 h‐laundry effect by cholesterol (+) liposomes also induces these spheroids to uptake both hydrophilic Sulforhodamine B (red) and hydrophobic Coumarin‐6 (green) significantly more compared to No treat under imaging with visualization of nuclei (blue). Moreover, the laundry effect facilitates the uptake of the hydrophobic one in the spheroids significantly more than the hydrophilic one by quantitative image analysis. Scale bar = 200 µm. Data = mean ± SEM. N = the number of dots as independent replicates in each graph. Statistical significance is denoted by ^*^ *p* < 0.033, ^**^ *p* < 0.002, ^***^ *p* < 0.001, and not significant (ns).

Membrane cholesterol hinders the penetration of hydrophobic molecules through lipidic aggregation. Therefore, cholesterol laundry of the membrane by cholesterol (+)‐liposomes is expected to facilitate the uptake of hydrophobic molecules more effectively than hydrophilic ones (Figure 2A). As validation, Hep3B cells take up significantly more hydrophobic Coumarin‐6 (green) than hydrophilic Sulforhodamine B (red) over 4 h after laundry by the cholesterol (+)‐liposomes for 24 h (Figure 2B).

In addition to the cell culture model, a lump model of HCC is produced by first harvesting cancer tissues from patients upon surgical dissection, followed by isolation and primary culture of patient cancer cells from the tissues. Cell spheroids are then formed in AggreWell plates, which are loaded and cultured in 3D microchannel network chips under continuous perfusion without (No treat) or with cholesterol (+)‐liposomes treatment (Figure 2C). As the cholesterol level is determined under imaging with nucleus identification, cholesterol (+) liposomes significantly reduced the level compared to No treat, validating the exertion of the diffusive laundry effect into the spheroids (Figure 2D). The 24 h‐laundry effect with cholesterol (+) liposomes also significantly improves the uptake of both hydrophilic Sulforhodamine B and hydrophobic Coumarin‐6 in the spheroids compared to No treat, as assessed by imaging with visualization of nuclei (Figure 2E). Furthermore, the laundry effect facilitates the uptake of the hydrophobic molecule in the spheroids significantly more than the hydrophilic one by quantitative image analysis.

### 2.3 HCC Patient Tissues in 3D Perfusion Chips to Validate Laundry Effect and Drug Uptake

As hypoxia is intensified by depleting O_2_ during liver cancer cell culture, the membrane level of cholesterol increases (Figure S3A). Consistently, when Hep3B cells are cultured in hypoxia (1% O_2_) for 48 h, cholesterol accumulation with Filipin staining increases significantly compared to normoxia, as shown by confocal imaging with quantitative analysis (Figure S3B).

**FIGURE 3:**
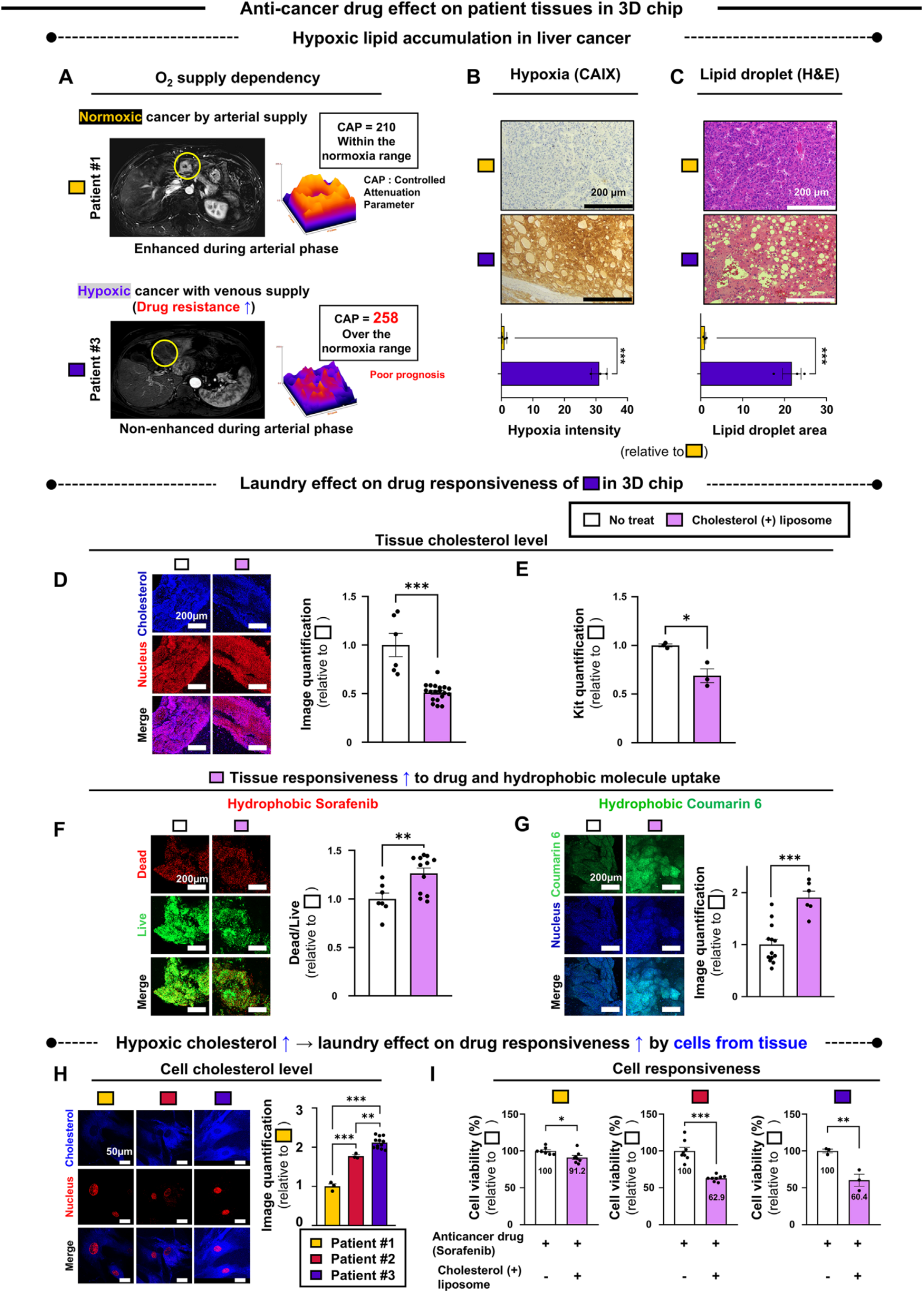
HCC patient tissues in 3D perfusion chips to validate cholesterol laundry effect and anti‐cancer drug responsiveness. (A) When MRI‐arterial phase imaging is carried out, the O_2_ richness under arterial supply serves as a classification point of HCC patient livers within or over the normoxic range (CAP: controlled attenuation parameter). The normoxic type (Patient #1) enhances the contrast of arterial phase upon sufficient vascularization as opposed to the hypoxic one (Patient #3) which is resistant to anti‐cancer drugs with poor prognosis. (B) The hypoxic cancer status of patient #3 liver is confirmed by the marker expression of CAIX which is significantly higher compared to patient #1 as assessed by the images (top) and quantitative image analysis (down). (C) Lipid accumulation with white vacuoles also increases significantly in the hypoxic cancer liver of patient #3 compared to patient #1 as assessed by H&E staining (top) with quantitative image analysis of the lipid droplet area (down). (D) A tissue piece (1.5 mm) of hypoxic liver cancer (patient #3) is obtained using a biopsy punch and cultured in 3D channel chip. The pre‐treatment of cholesterol (+)‐liposomes for 24 h significantly reduces the tissue cholesterol level (blue) compared to No treat in the confocal images (left, red nuclei) with quantitative analysis (right). (E) This result is confirmed using a biochemical kit assay. (F) The pre‐treatment of cholesterol (+)‐liposomes for 24 h significantly improves the responsiveness to hydrophobic Sorafenib (20 µm) in the hypoxic #3 tissue under 3D chip culture. This is indicated by a significant increase in the dead (red)/live (green) signals compared to No treat in the confocal images (left) with quantitative analysis (right). (G) The pre‐treatment of cholesterol (+)‐liposomes for 24 h significantly improves the uptake of hydrophobic coumarin‐6 (green) molecule by hypoxic #3 tissue (blue nuclei) in the 3D chip compared to No treat under confocal imaging (left) with quantitative analysis (right). (H) When cancer cells (red) are isolated from three Patient livers (#1–3), the membrane cholesterol level (blue) of normoxic #1 cells is significantly lower than those of mid‐hypoxic #2 and hypoxic #3 cell groups under confocal imaging (left) with quantitative analysis (right). (I) After the 24‐h pre‐treatment of cholesterol (+)‐liposomes, these three groups (#1–3) of patient cells significantly improve the responsiveness to the anti‐cancer drug (Sorafenib), as shown by the significant decreases in the cell viability compared to No treat. The anti‐cancer responsiveness of mid‐hypoxic (#2) and hypoxic (#3) cells are enhanced significantly more compared to normoxic (#1) cells. Together, as the membrane cholesterol level of HCC cells increases, the laundry effect and consequent drug responsiveness are improved accordingly. Statistical tests and exact sample sizes are indicated in each figure. Scale bar = 200 µm except 50 µm (H). Data = mean ± SEM. N = the number of dots as independent replicates in each graph. Statistical significance is denoted by ^*^ *p* < 0.033, ^**^ *p* < 0.002, ^***^ *p* < 0.001, and not significant (ns).

When MRI‐arterial phase imaging is performed, O_2_ richness under arterial supply serves as a classification criterion for HCC patient livers within or above the normoxic range (CAP: controlled attenuation parameter). The normoxic type (Patient #1) shows enhanced arterial phase contrast upon sufficient vascularization, in contrast to the mid‐hypoxic (Patient#2) and hypoxic (Patient #3) cases, which are resistant to anti‐cancer drugs and carry a poor prognosis (Figure 3A and Figure S4A). The incremental hypoxic status of patient #2 and #3 cancer livers is confirmed by significantly higher CAIX marker expression compared to patient #1, as assessed by imaging and quantitative image analysis (Figure 3B and Figure S4B). Lipid accumulation with white vacuoles also increases significantly in mid‐hypoxic (Patient #2) and hypoxic (Patient #3) cancer livers compared to normoxic patient #1, as assessed by H&E staining with quantitative image analysis of the lipid droplet area (Figure 3C and Figure S4C).

A tissue piece (1.5 mm) of hypoxic liver cancer (Patient #3) is obtained using a biopsy punch and cultured in a 3D channel chip. Pre‐treatment with cholesterol (+)‐liposomes for 24 h significantly reduces the tissue cholesterol level (blue) compared to No treat in confocal images with quantitative analysis (Figure 3D), and this result is confirmed by biochemical kit assay (Figure 3E). The 24 h pre‐treatment of cholesterol (+)‐liposomes also significantly improves responsiveness to hydrophobic Sorafenib (20 µm) in the hypoxic #3 tissue under 3D chip culture (Figure 3F), as indicated by a significant increase in dead /live signals compared to No treat in confocal images with quantitative analysis. Additionally, the pre‐treatment of cholesterol (+)‐liposomes for 24 h significantly improves the uptake of the hydrophobic coumarin‐6 (green) molecule by hypoxic #3 tissue in the 3D chip compared to No treat under confocal imaging with quantitative analysis (Figure 3G).

When cancer cells (red) are isolated from the three patient livers (#1‐3) (Figure 3H), the membrane cholesterol level (blue) of normoxic #1 cells is significantly lower than those of mid‐hypoxic #2 and hypoxic #3 cell groups, as assessed by confocal imaging with quantitative analysis. After 24‐h pre‐treatment of cholesterol (+)‐liposomes, all three patient cell groups (#1‐3) show significantly improved responsiveness to anti‐cancer Sorafenib, as evidenced by significant decreases in cell viability compared to No treat (Figure 3I). The anti‐cancer responsiveness of mid‐hypoxic (#2) and hypoxic (#3) cells is enhanced significantly more than that of normoxic (#1) cells. Together, as the membrane cholesterol level of HCC cells increases, both the laundry effect and consequent drug responsiveness are improved accordingly.

### 2.4 Cholesterol Laundry Therapy of Mouse Fatty Livers Against Pathogenesis

**FIGURE 4:**
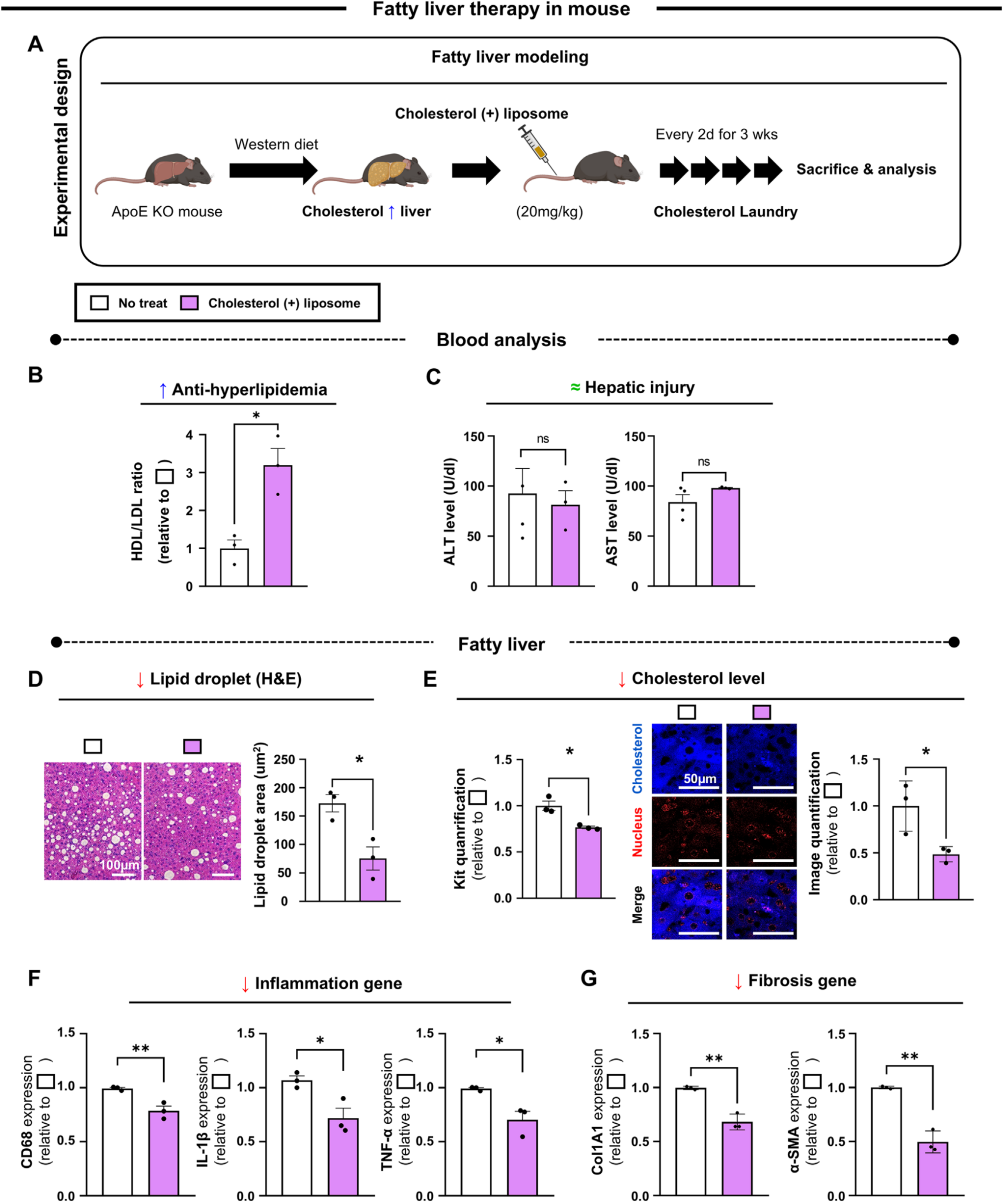
Cholesterol laundry therapy of mouse fatty livers against pathogenesis. (A) A mouse model of fatty liver is produced by subjecting ApoE KO mice to western diet for 7 weeks and then injection of cholesterol (+)‐liposomes (20 mg/kg) into tail veins every two days for 3 weeks, followed by sacrifice and analysis. (B) Compared to No treat, the IV injection of cholesterol (+)‐liposomes increases significantly the HDL/LDL ratio in blood compared to No treat, indicating an anti‐hyperlipidemia effect (H/LDL: high/low density lipoprotein). (C) Cholesterol (+)‐liposomes do not induce hepatic injury as the levels of ALT and AST are not significantly different from those of No treat (ALT: alanine aminotransferase /AST: aspartate aminotransferase). (D) Cholesterol (+)‐liposomes also reduce significantly formation of lipid droplets in mouse livers compared to No treat in the H&E images (left) and quantitative analysis (right). (E) This result is supported by significant reductions in the cholesterol levels of liver tissues by treatment of cholesterol (+)‐liposomes compared to No treat as double‐checked by a biochemical kit assay (left) and confocal imaging (middle, blue cholesterol and red nuclei) with quantitative analysis (right). (F) Cholesterol (+)‐liposomes mitigate significantly gene expression of inflammatory markers compared to No treat in qRT‐PCR analyses. The marker genes include CD 68 (macrophage infiltration), IL‐1β (Kupffer cell), and inflammatory cytokine (TNF‐α), whose expression is normalized to that of GAPDH. (G) Subsequently, the gene expressions of fibrosis markers (fibrous collagen protein: Col1A1 and hepatic stellate cell activation: α‐SMA) are reduced by cholesterol (+)‐liposomes compared to No treat upon normalization to that of GAPDH. A scale bar is denoted in each figure. Data = mean ± SEM. N = the number of dots as independent replicates in each graph. Statistical significance is denoted by ^*^ *p* < 0.033, ^**^ *p* < 0.002, ^***^ *p* < 0.001, and not significant (ns).

A mouse model of fatty liver is produced by subjecting ApoE KO mice to a western diet for 7 weeks, followed by tail vein injection of cholesterol (+)‐liposomes (20 mg/kg) every two days for 3 weeks, followed by sacrifice and analysis (Figure 4A). DiD‐labeled liposomes are injected via two clinical routes for 24 h to compare liver targeting in C57BL/6 mice so that the fatty liver therapy can be supported (Figure S5). IV injection significantly increases liposome accumulation in the liver as well as the spleen compared to IP injection, as assessed by IVIS imaging after harvesting the organs with quantitative analysis. Lung, kidney, and intestine show no significant differences in liposomal accumulation between the two groups.

Compared to No treat, IV injection of cholesterol (+)‐liposomes significantly increases the HDL/LDL ratio in blood, indicating an anti‐hyperlipidemia effect (H/LDL: high/low density lipoprotein) (Figure 4B). Cholesterol (+)‐liposomes do not induce hepatic injury, as the levels of ALT and AST are not significantly different from those of No treat (ALT: alanine aminotransferase /AST: aspartate aminotransferase) (Figure 4C). Cholesterol (+)‐liposomes also significantly reduce lipid droplet formation in mouse livers compared to No treat in the H and E images and quantitative analysis (Figure 4D). This result is supported by significant reductions in the cholesterol levels of liver tissues by the treatment of cholesterol (+)‐liposomes compared to No treat, as double‐checked by both biochemical kit assay and confocal imaging with quantitative analysis (Figure 4E).

Cholesterol (+)‐liposomes significantly mitigate the gene expression of inflammatory markers compared to No treat in qRT‐PCR analyses (Figure 4F). The marker genes include CD 68 (macrophage infiltration), IL‐1β (Kupffer cell), and the inflammatory cytokine (TNF‐α), whose expression is normalized to that of GAPDH. Subsequently, the gene expression of fibrosis markers (fibrous collagen protein: Col1A1 and hepatic stellate cell activation: α‐SMA) is also reduced by cholesterol (+)‐liposomes compared to No treat upon normalizing to that of GAPDH (Figure 4G).

### 2.5 Intracellular Metabolic Clearance of Membrane Cholesterol Through ER

**FIGURE 5:**
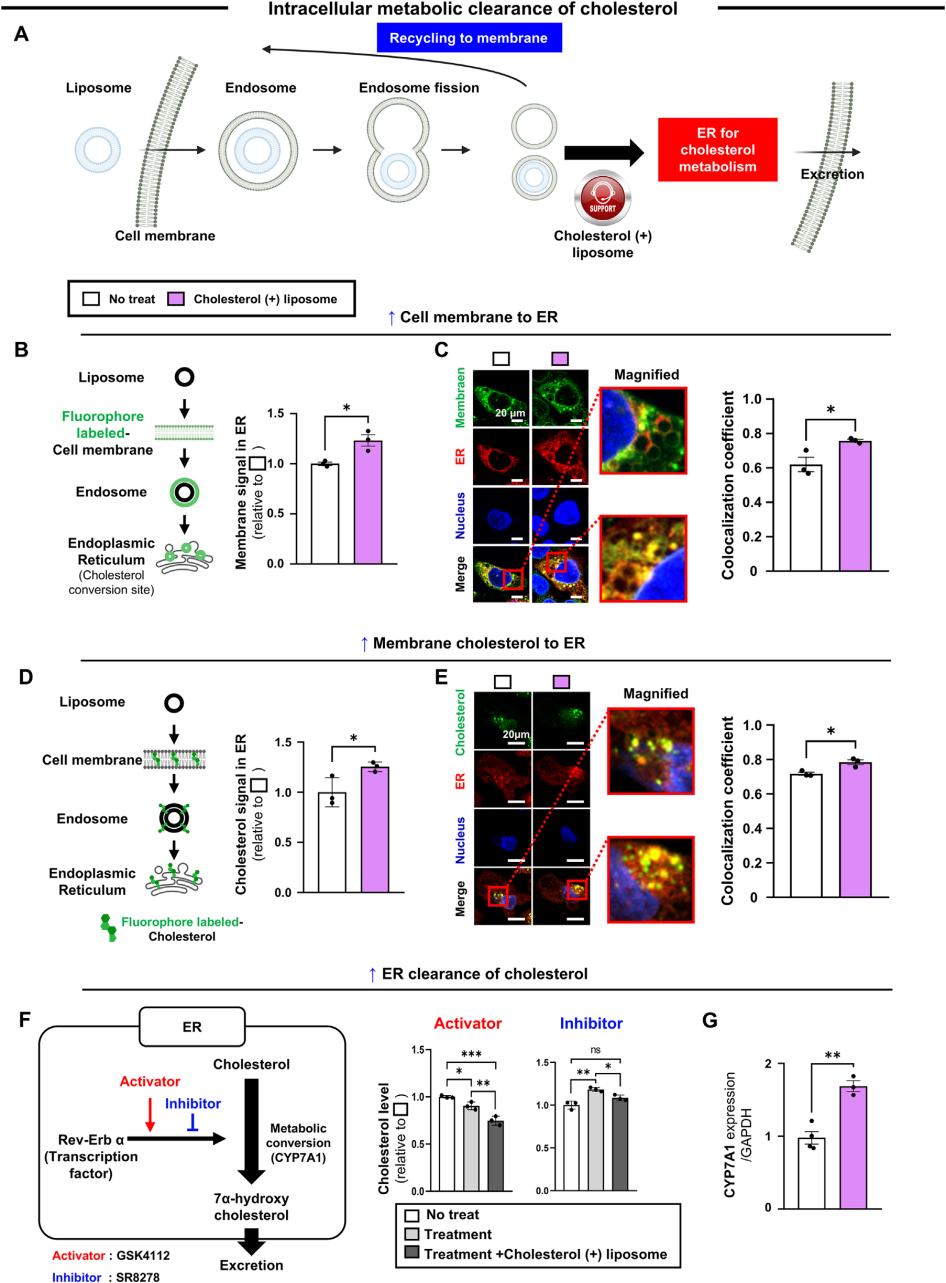
Intracellular metabolic clearance of membrane cholesterol through ER (A) The cholesterol (+)‐liposome penetrates the cholesterol‐rich membrane into plasma, and then, the membrane forms a cholesterol‐rich endosome by encapsulating the liposome. While cholesterol is transferred to the liposome, the endosome undergoes fission to either recycle back into the membrane without the liposome or carry the liposome into ER for metabolism, followed by excretion. (B) When the cell membrane is labeled with DiO (green) and subjected to treatment of cholesterol (+)‐liposomes, the transfer of DiO membrane to ER is determined after endosome formation by carrying liposomes (left). In the ER fractions upon isolation, the DiO intensity significantly increases by the liposome treatment compared to No treat upon microplate reading (right). (C) This result is confirmed by confocal imaging of the colocalization between the green DiO membrane and red ER around the blue nuclei (left), followed by the quantitative analysis of colocalization coefficient (right). These results indicate that the liposome treatment triggers the membrane transfer to ER where cholesterol is converted for metabolization. (D) When green BODIPY‐cholesterol is inserted into the cell membrane, which is subjected to treatment of cholesterol (+)‐liposomes, transfer of BODIPY‐cholesterol to ER is determined after endosome formation by carrying liposomes (left). In ER fractions upon isolation, the BODIPY intensity significantly increases by the liposome treatment compared to No treat upon microplate reading (right). (E) This result is confirmed by confocal imaging of colocalization between the green BODIPY‐cholesterol and red ER around blue nuclei (left), followed by quantitative analysis of the colocalization coefficient (right). These results indicate that the liposome treatment triggers membrane cholesterol to ER for conversion to be metabolized. (F) ER processes cholesterol metabolism by converting it to a relatively more water‐soluble form (7ɑ‐hydroxyl) through a key action of the transcription factor (Rev‐Erb ɑ) with expression of CYP7A1 (left). The level of membrane cholesterol increases in Hep3B cells under hypoxia culture (right), and Rev‐Erb ɑ activity is first activated by treating GSK4112 (20 µm). Consequently, the membrane cholesterol level decreases significantly from the co‐treatment with cholesterol (+) liposomes for 24 h to treatment of the activator only and further to No treat. When the Rev‐Erb ɑ activity is inhibited by treating SR8278 (20 µm), the membrane cholesterol level after the liposome co‐treatment results in non‐significant difference with the No treat level, and these two groups exhibit significantly lower levels than that of inhibitor only. The results indicate that the liposome treatment triggers cholesterol metabolism in ER through the action of Rev‐Erb ɑ as a key transcription factor. (G) This result is confirmed as the liposome treatment increases significantly the gene expression of CYP7A1with converting to water‐soluble 7ɑ‐hydroxyl cholesterol compared to No treat. Scale bar = 20 µm. Data = mean ± SEM. N = the number of dots as independent replicates in each graph. Statistical significance is denoted by ^*^ *p* < 0.033, ^**^ *p* < 0.002, ^***^ *p* < 0.001, and not significant (ns).

The cholesterol (+)‐liposome penetrates the cholesterol‐rich membrane into the plasma, after which the membrane forms a cholesterol‐rich endosome by encapsulating the liposome (Figure 5A). While cholesterol is transferred to the liposome, the endosome undergoes fission to either recycle back into the membrane without the liposome or to carry the liposome into the ER for metabolism, followed by excretion. When the cell membrane is labeled with DiO (green) and subjected to cholesterol (+)‐liposome treatment, transfer of the DiO membrane to the ER is determined following endosome formation by carrying liposomes (Figure 5B). In the ER fractions upon isolation, DiO intensity increases significantly with liposome treatment compared to No treat, as measured by microplate reading. This is confirmed by confocal imaging of colocalization between the green DiO membrane and the red ER around blue nuclei, followed by quantitative analysis of the colocalization coefficient (Figure 5C). These results indicate that the liposome treatment triggers membrane transfer to ER, where cholesterol is converted for metabolization.

When green BODIPY‐cholesterol is inserted into the cell membrane, which is subjected to the treatment of cholesterol (+)‐liposomes, the transfer of BODIPY‐cholesterol to the ER is determined following endosome formation by carrying liposomes (Figure 5D). In ER fractions upon isolation, BODIPY intensity increases significantly with liposome treatment compared to No treat upon microplate reading. This is confirmed by confocal imaging of colocalization between the green BODIPY‐cholesterol and the red ER around blue nuclei, followed by quantitative analysis of the colocalization coefficient (Figure 5E). These results indicate that the liposome treatment triggers the membrane cholesterol to the ER for conversion and metabolization.

To further verify the direct cholesterol transfer from the endosomal membrane to the liposome, a FRET acceptor photobleaching assay was performed using BODIPY‐cholesterol as the donor and DiI‐labeled cholesterol (+)‐liposomes as the acceptor. The principle of this assay is illustrated in Figure S6A. When the donor and acceptor reside within ∼10 nm, FRET occurs, and subsequent photobleaching of the acceptor abolishes the energy transfer, thereby restoring (increasing) the donor emission [22]. Therefore, an increase in donor signal upon acceptor photobleaching serves as direct evidence of close molecular proximity between the two fluorophores. Time‐lapse confocal imaging acquired every 5 s revealed that, upon photobleaching of DiI at 10 s, the green BODIPY‐cholesterol fluorescence increased while the red DiI fluorescence disappeared in the region of interest (Figure S6B), as further quantified by the time‐dependent intensity profiles of the donor and acceptor (Figure S6C). These results confirm that endosomal cholesterol is transferred to the liposome through direct molecular proximity within the endosomal compartment.

The ER processes cholesterol metabolism by converting it to water‐soluble 7ɑ‐hydroxyl cholesterol through the key action of transcription factor (Rev‐Erb ɑ) and the expression of CYP7A1 (Figure 5F). Under hypoxia culture, the membrane cholesterol level increases in Hep3B cells, and Rev‐Erb ɑ activity is first activated by treating with GSK4112 (20 µm). The membrane cholesterol level then decreases significantly from the co‐treatment with cholesterol (+) liposomes for 24 h to treatment of activator only, and further to No treat. When Rev‐Erb ɑ activity is inhibited by treating SR8278 (20 µm), the membrane cholesterol level following liposome co‐treatment results in no significant difference with the No treat level, and both of these groups exhibit significantly lower levels than that of the inhibitor only. These results indicate that the liposome treatment triggers cholesterol metabolism in the ER through the action of Rev‐Erb ɑ as a key transcription factor. This is further confirmed as the liposome treatment significantly increases the gene expression of CYP7A1with conversion to water‐soluble 7ɑ‐hydroxyl cholesterol compared to No treat (Figure 5G). Together, these results validate the key role of the ER with CYP7A1 and Rev‐Erb ɑ in cholesterol metabolism following endosomal transport of liposomes. To further validate the involvement of Rev‐Erbα and CYP7A1 in liposome‐mediated cholesterol metabolism, siRNA‐based knockdown experiments are conducted. The knockdown efficiency is confirmed by significant reductions in NR1D1 (encoding Rev‐Erbα) and CYP7A1 mRNA expression following treatment with their respective siRNAs compared to scramble siRNA (Figure S7A). Knockdown of NR1D1 significantly elevates the membrane cholesterol level compared to scramble siRNA (Figure S7B). A consistent trend is observed upon CYP7A1 knockdown. These results reinforce the role of Rev‐Erbα and CYP7A1 in regulating cholesterol metabolism.

## 3 Discussion

Drug efficacy is dependent on targeting efficiency through improved accuracy [23], arrival concentration through reduced clearance rate, and circulation route through selection of the injection site [24]. Continuous progress has been made not only in improving efficacy by controlling these factors, but also in developing smart delivery vehicles using advanced nanotechnologies [25, 26]. These efforts are aimed at improving drug efficacy without altering the cell status under targeting. Nevertheless, an unmet need remains to improve cellular uptake by reducing the barrier function of target cells, as drug penetration across the plasma membrane plays a key role in improving therapeutic efficiency [27]. In this regard, this study offers a meaningful solution to reduce the barrier function in pathogenic cells exhibiting elevated cholesterol levels in the membranes. The core strategy is to apply the concept of detergent to remove lipidic stains during laundry.

The amphiphilic nature of the plasma membrane makes it susceptible to cholesterol deposition, as hydrophilic phosphates adsorb water in a mixture of lipophilic molecules, which are simultaneously anchored into the lipid bilayer through hydrophobic interactions upon phase separation [15, 28]. Detergents are also amphiphilic, as they should be dissolved in water and capture lipidic stains in the same way to deposit cholesterol into the plasma membrane [29]. Liposomes are therefore used as a detergent template, owing to their shared amphiphilic components with the plasma membrane, in addition to the proven safety and effectiveness demonstrated in the real‐world clinic of COVID 19 to deliver mRNA vaccines into cells from circulation by IV injection [30, 31]. The detergent function is improved by inserting cholesterol into the liposomal membrane, which shows the maintenance of cell viability under increasing liposome concentrations, in contrast to the addition of Triton X‐100. This cholesterol insertion in the liposomal membrane is strategized to more effectively facilitate cholesterol transfer from the plasma to the liposomal membrane through hydrophobic interactions. Furthermore, regardless of the two membrane origins, cholesterol can be metabolized in the ER to excrete into the extracellular sides without any residual accumulation, which address concerns associated with other lipophilic detergents that may disturb cell function through residual effects [32].

HCC and fatty livers are chosen to test the detergent function of cholesterol (+)‐liposomes with laundry effects, given that lipid accumulation plays a central role in fatty liver formation. This pathogenesis drives HCC development through a stepwise progression from inflammation to fibrosis and further to NASH [33]. More importantly, any circulatory nanocarriers are dominantly accumulated in the liver for clearance, indicating natural hepatic targeting of liposomes even without optimization of targeting accuracy [34]. The spleen also serves as a major clearance site, though nanoparticles can exit the organ through capture and transport by splenic myeloid cells. This study demonstrates successful laundry effects and consequent drug uptake in hepatic tissues and cells from HCC patients. As hypoxic hepatic status increases in HCC patients, cholesterol level increases, providing additional justification for focusing on the liver for the present tests [35]. Even in the mouse fatty liver model, IV injection of cholesterol (+)‐liposomes alone results in attenuation of serial pathogenic events, from cholesterol laundry to inflammation and further to fibrosis. These results support cholesterol laundry therapy as a promising clinical approach while addressing concerns about nanotoxicity and side effects.

Cholesterol in the plasma membrane is amphiphilic, compromising hydroxyl and hydrocarbon groups in addition to four fused hydrocarbon rings. Consequently, the cholesterol is positioned from hydrophilic surface to the inner lipid layer of the plasma membrane, which indicates the amphiphilic nature of barrier function [36]. This rationale supports the results that the laundry of plasma membrane cholesterol promoted uptake of not only hydrophobic molecules but also hydrophilic ones. Moreover, when the decrease of membrane cholesterols disrupts lipid rafting, P‐gp efflux pump can be displaced by non‐raft membrane fraction and internalized into cells. Consequently, ATP‐drug efflux becomes dysfunctional and causes over accumulation of intracellular drugs [37, 38], resulting in reductions of cell viability [39]. However, the cell viability was maintained in response to the laundry of membrane cholesterol (Figure 1E), indicating non‐significant effects of cholesterol laundry on the P‐gp efflux pump. On the other hand, the influx pump systems, such as Organic Anion Transporting Polypeptides (OATPs) might contribute to improvement of drug uptake in the hepatic subjects [40]. However, it was clear that the cholesterol laundry improved hepatic uptakes of molecules and drugs, indicating the dominant effects regardless of the OATP contribution.

Hydrophobic Coumarin 6 is more prone to the laundry effect than hydrophilic Sulforhodamine B, as membrane cholesterol preferentially impedes the passage of lipophilic molecules through hydrophobic interactions [5]. Nonetheless, treatment of cholesterol (+)‐liposomes more effectively enhances the cytotoxic effect of hydrophilic doxorubicin compared to cholesterol (‐)‐liposomes and No treat, indicating that opening the transmembrane path by laundering cholesterol facilitates the transport of hydrophilic ones. Further studies are therefore required to clarify this point further using the same structure and size of drugs that differ only in hydrophobicity. Additionally, the same sizes of cholesterol (−) and (+)‐liposomes need to be compared, as a slight increase in the size (103 nm) of cholesterol (+) one from that (98 nm) of cholesterol (−) might affect the laundry effect. A large animal model of fatty liver is also approached in the next step to provide translational therapeutic insight prior to moving to the clinic. More patient samples are expected to support the validation of the current results further. Together, the current study demonstrates the potential to improve the therapeutic effects on HCC and fatty liver using the liposome technology that has been widely applied clinically for decades.

The mechanistic basis of the present laundry strategy is supported by previous studies, which demonstrate that membrane cholesterols can condense lipid bilayers to hinder drug penetration, and hydrophobic lipids can be transferred from target cell membrane to liposomes as an indication of the laundry process [41]. On the other hand, some side effects are expected because when cholesterols are transferred to the liposomal membrane with consequent condensing of the lipid bilayer, payload release from the liposomes can be hindered [42]. When HCC decreases the membrane cholesterol, the efflux of ABCB1 pump is destabilized because of dysfunction in lipid rafting, indicating another contradictory effect. These points require further studies to improve the liposomal laundry strategy for clinical translation. These further studies should also increase the number of HCC patient samples and validate the long‐term safety profile by repeating IV administration in large animals.

This study did not scrutinize lysosomal entrapment versus cytosolic escape. After penetrating target cell membranes, liposomes can be trapped through the formation of endosomes and subsequent endolysosomes. Consequently, liposomal disruption is accelerated because of low pH (∼4.5) inside endolysosomes. For example, a study has reported that over 90% of liposomes to carry doxorubicin are trapped by endolysosomes, which limits cytosolic drug effects [43]. In this regard, a considerable solution can be approached to use endolysosomes as a remover of cholesterol instead of processing liposomal disruption using fusogenic lipids or pH‐responsive moieties [44]. Incorporation of these components into liposomes to lauder plasma membrane cholesterols accelerates liposomal fusion with endolysosomes or disruption to release the cholesterols into endolysosomes. In this way, cholesterol removal by the low pH in endolysosomes can be facilitated, although drug release from the liposomes is limited if strategized. The pros and cons of this approach represent another subject to study in the next stage.

The representative novelty of the present study lies in the concept of plasma membrane laundry by using cholesterol (+)‐liposomes as a therapeutic detergent because the membrane cholesterols act as a barrier to drug permeability. As a conclusion, this liposomal strategy enhanced consequent cellular uptake of drugs and molecules and reduced the lipid‐driven pathogenic events in HCC patient samples and mouse fatty livers, revealing therapeutic benefits. The favorable profiles of liposomal biosafety in addition to cholesterol clearance in the ER support the translational potential to the hepatic clinic.

In conclusion, this liposomal strategy enhanced consequent cellular uptake of drugs and molecules and reduced the lipid‐driven pathogenic events in HCC patient samples and mouse fatty livers, revealing therapeutic benefits. The favorable profiles of liposomal biosafety, in addition to cholesterol clearance in ER support the translational potential to the hepatic clinic.

## 4 Experimental Section

### 4.1 Liposome Preparation

Cholesterol (+)‐liposomes were prepared using the rapid ethanol injection method [32]. First, (i) dipalmitoyl phosphatidylcholine (850355P, Sigma–Aldrich, St. Louis, MO, USA), (ii) cholesterol (C8667, Sigma–Aldrich), and (iii) 1,2‐distearoyl‐sn‐glycero‐ 3‐phosphoethanolamine‐N‐[methoxy (polyethylene glycol)] (DSPE‐PEG, 880120P, Sigma–Aldrich) were mixed at a molar ratio of 55:40:5 in ethanol (E7023, Sigma–Aldrich) at 72°C for 5 min. The mixture was then rapidly injected into a fourfold volume of PBS using a needle syringe (20 G, Restek, Bellefonte, PA, USA) under stirring at 500 rpm at 72°C for 5 min, followed by adding an additional equal volume of PBS under vigorous stirring for 15 min at room temperature.

A filter system was set up by assembling the (top) drain disc (PETEDD9025; Sterlitech, Auburn, WA, USA), (middle) 0.1 µm membrane filter (PCT019030, Sterlitech), and (bottom) another drain disc, which was used to filter the mixture in PBS 11 times using an extruder (610 000, Avanti Polar Lipids, Inc., 700 Industrial Park Drive, Alabaster, AL 35007, USA). Through this process, the self‐assembly of liposomal components was processed to form micelles, and the liposomes were collected by centrifugation (26 000 × g) at 4°C for 30 min. Cholesterol (−)‐liposomes were produced following the same process without cholesterol. Detergent (+)‐liposomes were prepared by incubating Cholesterol (−)‐liposomes with Triton X‐100 (93443, Sigma–Aldrich) in PBS at room temperature for 30 min, followed by centrifugation (26 000 × g) for 30 min and pellet resuspension in PBS twice. Critical micelle concentration (CMC) of Triton X‐100 was tested in the range of 0.015%–0.06% (w/v) to prevent the lytic effect on the lipid bilayer. Triton X‐100 was applied at concentrations of 0.06, 0.13, 0.25, 0.5, and 1.0 mm, which corresponded to 0.004%, 0.008%, 0.016%, 0.032%, and 0.064% w/v, respectively.

### 4.2 Liposome Characterization

Liposomal concentration was determined using the Stewart assay. Dipalmitoylphosphatidylcholine (DPPC) was dissolved in chloroform (6955, Duksan, Ansan, Republic of Korea) at a concentration of 0.1 µg/mL to make a standard solution. A ferrothiocyanate reagent was prepared by dissolving ferric chloride hexahydrate (27.03 g, F2877, Sigma–Aldrich) and ammonium thiocyanate (30.4 g, 221988, Sigma–Aldrich) in 1 L of deionized water, which was then reacted with each liposome sample. The mixture was vortexed for 20 s and centrifuged at 300 × g for 10 min to induce phase separation, followed by reading the absorbance of the lower phase at 485 nm using an ELISA reader (VERSA Max, Molecular devices). The Triton X‐100 concentration in Detergent (+)‐liposome membranes was determined by recording the absorbance spectra of aromatic chromophore over 275–283 nm in a UV–vis spectrophotometer (Lambda25, PERKINELMER). The morphology and size distribution of each liposome sample were examined by transmission electron microscopy (TEM, Jem2100, JEOL, Tokyo, Japan) and nanoparticle tracking analysis (NTA, NS300, Malvern Panalytical, Malvern, UK), respectively.

### 4.3 Biosafety Assessments of Liposome

To ensure the hemocompatibility of cholesterol (+)‐liposome for systemic administration, a hemolysis assay was performed using red blood cells (RBCs) isolated from C57BL/6 mice. Whole blood was centrifuged (2000 × g) at 4°C for 20 min to collect the RBC fraction. To establish a standard for osmotic fragility, a serial dilution of NaCl solutions was prepared, ranging from 0% to 0.9%. Specifically, 100 µL of liposome mixtures (at final concentrations of 0, 0.2, 0.4, and 0.8 mg/mL) were added to 1 mL of each NaCl solution containing 5 µL of the isolated RBCs. The mixtures were then incubated at 4°C for 2 h to evaluate the extent of membrane disruption. Following incubation, the samples were centrifuged (800 × g) at 4°C for 3 min, and the absorbance of the supernatant was measured at 540 nm, the characteristic wavelength of hemoglobin, using a microplate reader.

Primary human umbilical vein endothelial cells were purchased from Lonza (HUVEC, C2519A, Basel, Switzerland; passage 4–8), using endothelial growth medium MV2 kit (EGM, C‐22022, PromoCell, Heidelberg, Germany). To evaluate the biosafety of the cholesterol (+)‐liposome, cytotoxicity was assessed in HUVECs as a model for healthy vascular endothelium.

HUVECs were seeded in 96‐well plates and treated with liposomes at varying concentrations (0, 0.2, 0.4, and 0.8 mg/mL) for 24 h. Cell viability was subsequently determined using a Cell Counting Kit‐8 (CCK‐8; Dojindo) according to the manufacturer's protocol, with absorbance measured at 450 nm using a microplate reader.

### 4.4 Hypoxic Cholesterol Elevation

As a cell line of hepatocellular carcinoma (HCC), Hep3B (passage 35–54) was purchased from the Korean Cell Line Research Foundation (88064, Seoul, Republic of Korea). Hep3B was cultured using DMEM (L0103‐500, Biowest, Kyungsan, Korea) with inactivated 10% fetal bovine serum (FBS, 16000–044, Gibco, Carlsbad, CA, USA) and 1% penicillin‐streptomycin (PS, 15140‐122, Invitrogen, Carlsbad, CA, USA) at 37°C with 20% O_2_ and 5% CO_2_. The cholesterol level of the cell membrane was increased by exposing Hep3B to 1% O_2_ for 48 h in a hypoxia incubator (4131, Thermo Fisher, Waltham, MA, USA).

### 4.5 HCC Patient Tissue

Three HCC patients provided consent and donated liver cancer tissue following surgical removal, under protocols approved by the Institutional Review Board (IRB) of Yonsei University College of Medicine (IRB: 4‐2016‐0728). These patients were diagnosed at early HCC stages according to the Barcelona Clinical Liver Cancer system. While patient #1 exhibited uniform enhancement in the arterial phase above the average level of livers, patients #2 and #3 displayed no enhancement, with only a visible peripheral border and rim center. The MRI contrast intensity of each patient's liver was quantitatively analyzed by making a 3D surface plot plugin using ImageJ (Fiji). Diagnostic information of each patient was analyzed, including fatty liver disease (CAP), tumor marker levels (AFP, PIVKA‐II), liver function levels (AST, ALT, D. Bilirubin), and glucose. Following surgical removal within two months of diagnosis, liver cancer tissues were analyzed histologically to examine tumor volume and tissue necrosis (Table 2). These tissues were immediately delivered to the laboratory in cold culture media.

When cells were harvested, these tissues were minced using grinder tips (G50; Coyote Bioscience, BJ, China) and centrifuged at 300 × g for 3 min. Tissue pieces were then incubated with collagenase type IV (200 UI/mL, 17104019, Gibco) in Hank's balanced salt solution (HBSS) at 37°C for 1 h under shaking (200 rpm). The solution was filtered through the 70 µm cell strainer (93070, SPL) to collect cells, and the collagenase was deactivated by washing three times using DMEM with 10% FBS. The cells were then cultured in DMEM with 10% FBS, 1% PS (15140‐122, Gibco) for 5–10 passages at 37°C with 20% O_2_ and 5% CO_2_.

### 4.6 Cell Spheroid

Cell spheroids were generated using AggreWell 800 plates (STEMCELL Technologies, #34811) following the manufacturer's protocol. Briefly, each well was treated with 500 µL of anti‐adherence rinsing solution and centrifuged at 1300 × g for 5 min to remove air bubbles from the microwells and washed with basal medium (2 mL/well). Cells were seeded at a density of 5 × 10^5^ cells per well in 2 mL of medium and centrifuged at 100 × g for 3 min to facilitate uniform cell aggregation.

### 4.7 3D Channel Chip

Cell spheroids and patient tissues were cultured in 3D gelatin chips with microchannel networks under continuous media perfusion. First, as a source of microchannel networks, sacrificial microfibers were produced by melt‐spinning a PCL–PVAc–PEG copolymer (Soluplus, n30446233; BASF, Ludwigshafen, Germany) in 66% (w/v) methanol solution using a custom‐built spinning device. The diameter range of the microfibers was controlled by adjusting the rotational speed of the spinning device (140–176 × g). Then, a density range (0–3 mg/mL) of fibers was placed into a mold (10 mm‐diameter and 2 mm‐height) of polydimethylsiloxane (PDMS, 31‐000810‐02; Dow Corning, Midland, MI, USA). The bottom of the PDMS mold was sealed onto a glass slide (1000412; Marienfeld, Lauda‐Königshofen, Germany). Perfusion inlet and outlet ports were generated in 1.5 and 1.0 mm in diameters, respectively, by piercing the PDMS mold with biopsy punches of (BPP‐15F and BPP‐10F; KAI Industries).

Microfibers were then stacked to encapsulate cell spheroids or patient tissues in the PDMS mold, enabling sufficient media perfusion for oxygen and nutrient supply. Gelatin solution (5.5% w/v, G1890; Sigma‐Aldrich) was poured into the mold to embed the samples and fibers. A hydrogel was produced by crosslinking the gelatin with microbial transglutaminase (1% w/v, mTG, 1201–50; Modernist Pantry LLC, Eliot, ME, USA) at a volume ratio of 9:1 at 37°C for 30 min. The sacrificial microfibers were subsequently removed via a gel‐to‐solution transition at room temperature, below the lower critical solution temperature (LCST) of Soluplus (38°C) to generate microchannels with thorough perfusion using PBS. Luer tubes were inserted into the inlet port so that media could be perfused into the inlet, circulated throughout the microchannel network, and exit through the outlet. Media perfusion was maintained at a flow rate of 160 µL/min using a peristaltic pump (BT100‐1L; Longer Precision Pump, Amersham, UK).

### 4.8 Cholesterol Quantification

Cholesterol levels were quantified using a Cholesterol Quantification Assay Kit (ab102515, Abcam) according to the manufacturer's instructions. Cells (1 × 10^6^) or tissue samples (10 mg) were lysed in 200 µL of chloroform:isopropanol:NP‐40 (7:11:0.1, v/v) using a micro‐homogenizer. Lipid components were collected as the supernatant after centrifuging the lysates at 15 000 × g for 5 min, air‐dried at 60°C, and resuspended in 200 µL of Assay Buffer. Each sample (50 µL) was transferred to a 96‐well plate, the reaction mixture was added, and the samples were incubated at 37°C for 30 min. Absorbance was then recorded at 450 nm using a microplate reader, and the corresponding cholesterol concentration was calculated according to the kit protocol.

For imaging quantification, monolayer cells in culture were washed with PBS and fixed with 4% paraformaldehyde (PFA) for 5 min at room temperature, followed by three PBS washes. Cells were then incubated with Filipin complex (25 µg/mL, F9765, Sigma–Aldrich) in PBS:DMSO (4:1, v/v) for 2 h at 4°C in the dark. Cell spheroids or patient tissues from biopsy were cultured in a 3D micro‐channel network chip, fixed with 4% PFA for 30 min at room temperature, and washed with PBS three times for 10 min each. Chip samples were incubated with Filipin solution (50 µg/mL) for 4 h at 4°C in the dark. Mouse liver tissues were harvested and washed three times with cold PBS to remove residual host blood. Samples were embedded in frozen section compound (3801480, Leica Biosystems) and stored at −80°C, followed by sectioning to 4 µm thickness using a cryostat (CM1860; Leica Biosystems). After air‐drying on glass slides for 30 min at room temperature, sections were fixed with 4% PFA in PBS for 10 min at room temperature, rinsed three times with PBS, and incubated with Filipin solution (50 µg/mL) for 4 h at 4°C. After washing three times with PBS, cell nuclei were counterstained with NucRed Live 647 ReadyProbes reagent (R37106; Invitrogen). After adding aqueous mounting medium (Glycerin jelly, 17998‐10; Electron Microscopy Sciences, Hatfield, PA, USA), the sections were imaged under confocal microscopy (LSM 900, Zeiss, Zen 3.3 blue edition), followed by quantitative image analysis using ImageJ (Fiji).

### 4.9 Endosome Model Nanovesicle

As an endosome model, cell membrane–derived nanovesicles were produced from cholesterol‐rich Hep3B cells. Cells were first incubated in a hypotonic solution for 30 min on ice to induce membrane swelling and disruption, followed by filtration through the extrusion system without a membrane filter 100 times (see above). After centrifugation at 700 × g for 10 min, the supernatant was serially filtered from 5 µm to 0.4 µm three times per filter, and nanovesicles were collected by re‐centrifugation and resuspension in PBS. The surface marker CD133 and membrane cholesterol of the nanovesicles were labeled with a red fluorescence conjugate (ab226355, Abcam, Cambridge, UK) and BODIPY‐cholesterol (HY‐125746, MedChemExpress, Monmouth Junction, NJ, USA), respectively, following the suppliers’ protocols. The endosome nanovesicles were incubated with liposomes for 2 h at room temperature, followed by flow cytometric analysis with FlowJo.

### 4.10 Drug Uptake

As a model of HCC, Hep3B cell line (2 × 10^3^ cells per well) was cultured on 96‐well plates with 100 µL of medium in a hypoxia incubator to elevate membrane cholesterol levels. The cholesterol laundry effect was examined by treating the cells with test liposomes (400 µg/mL) for 24 h. Then, the drug responsiveness was determined by treating the cells with sorafenib (20 µm) in DMEM for 24 h at 37°C in 5% CO_2_, with cell viability determined using the Cell Counting Kit‐8 (CCK‐8; Dojindo). Briefly, CCK‐8 solution (10 µL/ well) was incubated for 30 min at 37°C, and absorbance was measured at 450 nm in a microplate reader. This assay was used to determine the cytotoxic liposomal concentration (0, 0.2, 0.4, and 0.8 µg/mL) on Hep3B.

After culturing in 3D chips under perfusion, spheroids and patient tissues were treated with test liposomes (400 µg/mL) for 24 h, followed by treatment with sorafenib (50 µm) in DMEM under continuous perfusion for 24 h at 37°C. Samples were fixed in 4% PFA for 30 min at room temperature and washed three times with PBS for 10 min each. Then, Calcein‐AM (0.5 µL) and EthD‐1 (2 µL) were treated in 1 mL PBS for 60 min at room temperature, followed by washing and confocal imaging (LSM 900, Zeiss) at excitation/emission wavelengths of 488/515 nm (Calcein‐AM) and 528/617 nm (EthD‐1).

After treating cholesterol (+)‐liposomes, Hep3B uptake of hydrophilic sulforhodamine B (20 µm, 230162; Sigma–Aldrich) was compared with that of hydrophobic Coumarin 6 (20 µm, 442631; Sigma–Aldrich) in DMEM for 6 h at 37°C, followed by washing with PBS. As both molecules are fluorescent, intracellular fluorescence intensity was quantified in a microplate reader (Varioskan LUX, Thermo Scientific) at 565/586 nm (Sulforhodamine B) and 488/505 nm (Coumarin 6). When cell spheroids or patient tissues were cultured in 3D chips and treated with cholesterol (+) liposomes under perfusion, 50 µm of sulforhodamine B or Coumarin 6 was treated for 6 h at 37°C under continuous perfusion. After washing, cell nuclei were counterstained with DAPI, and samples were imaged in z‐stacks by confocal microscopy, followed by quantitative image analysis using ImageJ (Fiji).

### 4.11 Animal Experiment

All animal experiments were conducted following the guidelines and protocols approved by the Institutional Animal Care and Use Committee of the Yonsei University College of Medicine (Permit No. 2024‐0321). Apolipoprotein E–deficient (ApoE^−^/^−^) mice were purchased from The Jackson Laboratory (Bar Harbor, ME, USA) and fed a western diet (RD Western Diet, D12079B; Research Diets, New Brunswick, NJ, USA) for 7 weeks to accumulate hepatic cholesterol. Mice were randomly grouped to No treat under saline injection (200 µL), and Cholesterol (+)‐liposome, which was injected at 20 mg/kg weight in saline (200 µL) via tail vein every 2 days for 3 weeks using an insulin syringe (29 G; U‐100; Terumo, Tokyo, Japan).

After anesthetizing mice, blood was drawn via cardiac puncture using a 1 mL syringe and centrifuged at 2000 × g for 10 min to obtain plasma. Then, DRI‐CHEM 4000i analyzer (FUJIFILM Corporation, Tokyo, Japan) with FUJI Dri‐Chem slides was used to analyze plasma biochemical parameters, including GOT/AST‐P III (#3150), GPT/ALT‐P III (#3250), TCHO‐P III (#1450), TG‐P (#1650), and HDL‐C‐P III (#2650). The levels of low‐density lipoprotein cholesterol (LDL‐C) were calculated using the Friedewald formula:

(1) $$\begin{array}{cc} & LDL-C\;(mg/dL)\\ & \;=TCHO\;(mg/dL)\;-\;[HDL-C\;(mg/dL)+TG\;(mg\;d/L)/5] \end{array}$$

### 4.12 Immunohistochemistry

Tissue samples were embedded in paraffin, sectioned to 4 µm‐thickness slices, and underwent Hematoxylin and eosin (H and E) staining using standard protocols, followed by inverted microscope imaging (DMi8 Leica, Wetzlar, Germany). Sections were deparaffinized with xylene twice for 10 min each and rehydrated using ethanol in a serial dilution with distilled water (100, 95%, 80%, and 70% v/v for 5 min each). Endogenous peroxidases were deactivated by incubation in 3% H2O_2_ solution (H1009, Sigma–Aldrich), followed by washing with tris‐buffered saline (ML023‐03, Welgene) and blocking with 3% bovine serum albumin (BSA, A0100‐005, GenDEPOT, Altair, TX, USA) in PBS. The sections were reacted with a primary antibody for CAIX (1:1000, NB100‐417, Novus Biological LLC) at RT for 1 h, and then with an HRP‐labeled secondary antibody (1:5000, anti‐rabbit polymer, k4003, Agilent Dako) at RT for 20 min. Next, the samples were subjected to treatment with DAB development solution (k3468, Agilent Dako) for 5 min, deionized water washing, hematoxylin counter‐staining (k8008, Agilent Dako), and optical imaging.

### 4.13 qRT‐PCR

Total RNA was extracted from each sample using Hybrid‐R (305‐101; GeneAll, Seoul, Republic of Korea), followed by reverse transcription of 1 µg of RNA into cDNA using PrimeScript RT Master Mix (RR036A; Takara, Kusatsu, Shiga, Japan) according to the manufacturer's instructions. The entire primer sequences were designed using the National Center for Biotechnology Information via Primer‐BLAST (Table 1), and the primer oligonucleotides were synthesized in Cosmogenetech (Seongdong‐gu, Seoul, Republic of Korea). Step‐One Real‐time PCR (Applied Biosystems, Foster City, CA, USA) was run for 40 cycles to amplify target genes using SYBR Green and cDNA primers. Gene expression levels were determined relative to the C_t_ value of glyceraldehyde 3‐phosphate dehydrogenase (GAPDH: housekeeping gene) using StepOnePlus version 2.3. Data were presented as dot blot graphs in relative C_t_ values to those of No treat.

**TABLE 1:** PCR primers.

Gene	Primer Sequence	Orientation
Mouse CD68	GGACCCACAACTGTCACTCAT	Forward
	AAGCCCCACTTTAGCTTTACC	Reverse
Mouse IL‐1β	GCAACTGTTCCTGAACTCAACT	Forward
	ATCTTTTGGGGTCCGTCAACT	Reverse
Mouse TNF‐ α	GACGTGGAACTGGCAGAAGAG	Forward
	TTGGTGGTTTGTGAGTGTGAG	Reverse
Mouse Col1A1	GCTCCTCTTAGGGGCCACT	Forward
	CCACGTCTCACCATTGGGG	Reverse
Mouse α‐SMA	CCCAACTGGGACCACATGG	Forward
	TACATGCGGGGGACATTGAAG	Reverse
Mouse GAPDH	GCG AGA CCC CAC TAA CAT CA	Forward
	GGC GGA GAT GAT GAC CCT TT	Reverse
Human CYP7A1	GAGGCACGAGAACCTCCAAA	Forward
	TGGAATGGTGTTTGCTTGCG	Reverse
Human NR1D1	ATT CCG AGA AGC TGC TGT CC	Forward
	GAA GTT CGG TGA TGG GGG AG	Reverse
Human GAPDH	TCA AGG CTG AGA ACG GGA AG	Forward
	CGC CCC ACT TGA TTT TGG AG	Reverse

### 4.14 Cholesterol Trafficking Post Uptake

The plasma membrane of Hep3B was labeled with Vybrant DiO Cell‐Labeling Solution (5 µL, V22886, Invitrogen), while BODIPY‐cholesterol (1 µm, HY‐125746, MedChemExpress) was inserted into the membrane to distinguish trafficking of the membrane from that of the membrane cholesterol. Both were labeled through incubation in DMEM (1 mL) for 30 min at 37°C in 5% CO_2_, and excess dyes were washed with PBS. Then, these cells were treated with cholesterol (+)‐liposomes (400 µg/mL) for 24 h. The plasma membrane was used to form endosomes by encapsulating the liposomes, so that cholesterol can be transferred from the plasma membrane to the endosomal membrane and further to the liposome membrane. Afterward, the endosome undergoes fission, either to move back into the cell membrane or to carry the liposomes to ER for clearance.

Hence, ER fractions were isolated using the Endoplasmic Reticulum Isolation Kit (ER0100, Sigma–Aldrich) following the manufacturer's protocol. Briefly, Hep3B cells (5 × 10^5^) were incubated in hypotonic extraction buffer on ice (4°C) for 20 min and centrifuged at 600 × g for 5 min. The supernatant was discarded, and the pellet was resuspended in isotonic buffer and homogenized using Dounce homogenizer. The homogenate was centrifuged at 1000 × g for 10 min at 4°C to remove nuclei and unbroken cells, and the supernatant was further centrifuged at 12 000 × g for 15 min at 4°C, with the final supernatant preserved as the ER fraction. The fluorescence signal from DiO‐ membrane or BODIPY‐cholesterol within the ER fraction was quantified in a microplate reader (Varioskan LUX, Thermo Scientific) at 484/501 nm (DiO excitation/emission) or 488/508 nm (BODIPY). Colocalization was examined by labeling ER with ER‐Tracker Red (1 µm, E34250, Invitrogen) in HBSS (LB003‐04, Welgene) for 30 min at 37°C in addition to DiO‐plasma and BODIPY‐cholesterol, followed by counter‐staining of nuclei with DAPI and confocal imaging (Zeiss LSM 900). Colocalization was quantified by calculating Manders’ overlap coefficients from background‐subtracted images.

To distinguish the physical transfer of cholesterol from the cell membrane to the liposomal bilayer, acceptor photobleaching fluorescence resonance energy transfer (FRET) imaging was performed. BODIPY‐cholesterol, as a donor fluorophore, was incorporated into the Hep3B cell membrane, while cholesterol (+)‐liposomes were stained with Vybrant DiI Cell‐Labeling Solution (V22885, Invitrogen), as an acceptor fluorophore. Hep3B cells were incubated with DiI‐stained cholesterol (+)‐liposomes (400 µg/mL) to allow for potential cholesterol exchange.

FRET analysis was conducted using a confocal microscopy (LSM 980, Zeiss), equipped with a 63x oil‐immersion objective.

Time‐lapse images were captured at 5 s intervals to monitor the fluorescence intensities of both the donor and acceptor. At the 10 s mark, a specific region of interest (ROI) containing the acceptor signals was selectively photobleached using high‐intensity 561 nm laser pulses. The subsequent recovery of the donor (BODIPY) fluorescence intensity was monitored in real‐time to validate the FRET effect, confirming the close proximity and physical transfer of membrane cholesterol to the liposomal bilayer Table 2.

**TABLE 2:** Clinical parameters of three patients.

Outcome (Patient)	Normoxic #1	Mid‐Hypoxic #2	Hypoxic #3
Tumor volume(cm^3^)	34	153	75
Necrosis (%)	10	30	70
AFP (ng/mL)	2.1	7557	5.5
PIVKA‐II (ng/mL)	78.6	887	53.8
AST (IU/L)	98	149	71
ALT (IU/L)	82	48	39
D. Bilirubin (mg/dL)	0.3	0.5	1.1
Glucose (mg/dL)	131	136	157

### 4.15 Cholesterol Clearance in ER

The ER processes cholesterol metabolism by converting to water‐soluble 7ɑ‐hydroxyl cholesterol through the key action of transcription factor (Rev‐Erb ɑ) and the expression of CYP7A1. This ER mechanism was validated by increasing the level of membrane cholesterol in Hep3B cells under hypoxia culture. Then, Rev‐Erb ɑ activity was inhibited by treating SR8278 (20 µm, 554718, Sigma–Aldrich) or activated by treating GSK4112 (20 µm, 554716, Sigma–Aldrich) with or without the treatment of cholesterol (+) liposomes for 24 h. The levels of membrane cholesterol were subsequently determined using a Cholesterol Quantification Assay Kit (ab102515, Abcam) according to the manufacturer's protocol.

To genetically validate the involvement of the Rev‐Erb ɑ/CYP7A1 axis in liposome‐mediated cholesterol metabolism, RNA interference (RNAi) was performed. The siRNAs were purchased from Bioneer (Daejeon, Republic of Korea) as AccuTarget Genome‐wide Predesigned siRNAs targeting *NR1D1* (Rev‐Erb ɑ) and *CYP7A1* or non‐targeting scramble siRNA. Hep3B cells were transfected with siRNAs targeting *NR1D1* (Rev‐Erb ɑ), *CYP7A1*, or a non‐targeting scramble siRNA using Lipofectamine RNAiMAX Transfection Reagent (13778075, Invitrogen) in Opti‐MEM reduced serum medium (31985070, Invitrogen) according to the manufacturer's instructions. After 24 h of transfection, the knockdown efficiency was confirmed at the mRNA via qRT‐PCR analysis. Following the confirmation of gene knockdown, cholesterol accumulated cells were treated for 24 h, prior to quantifying the cholesterol levels.

### 4.16 Liposome Distribution to Mouse Organs

Cholesterol (+) liposomes were labeled with Vybrant DiD Cell‐Labeling Solution (5 µL, V22887, Invitrogen) in 1 mL PBS for 30 min at 37°C in the dark, followed by PBS washing to remove excess dye. These liposomes were administered to mice at a dose of 20 mg/kg via either intraperitoneal or intravenous injection for 24 h. After the mice were sacrificed by CO_2_ inhalation, organs were harvested, and their fluorescence signals were measured using an in vivo imaging system (IVIS, 124 262, PerkinElmer) at 644/665 nm (DiD excitation/emission).

### 4.17 Statistical Analysis

Data were analyzed using Prism 10.1.2 (GraphPad Software, Boston, MA, USA) and Excel (version 16.0.17531.20004) with presentation as means ± standard error of the mean. Paired comparisons were assessed using a two‐tailed Student's t‐test, while multiple comparisons were performed with one‐way ANOVA with Tukey's significant difference post‐hoc test. Statistically significant values were indicated as *p*‐value ≤ 0.033 = ^*^, < 0.002 = ^**^, < 0.001 = ^***^. As biological replicates, n numbers were described in the figure legends.

## Author Contributions

H.‐J.S. conceived the study. C.K., J.K.N., and S.B. designed the experiments. C.K., J.K.N., S.B., S.E.Y., J.K., S.L., and Y.O. performed the experiments. S.C. and D.H.H. developed the reagents and analytical tools necessary for the study. D.H.H. provided clinical samples. J.K.N. and S.B. conducted the data analysis. C.K., J.K.N., and S.B. drafted the initial version of the manuscript. C.K., J.K.N., S.B., and H.‐J.S. analyzed the data. H.‐J.S. supervised the study and provided critical revisions of the manuscript. All authors read and approved the final manuscript.

## Conflicts of Interest

The authors declare no conflicts of interest.

## Supporting information




**Supporting File**: advs76144‐sup‐0001‐SuppMat.docx.

## Data Availability

The data that support the findings of this study are available in the supplementary material of this article.
