# Supplementary material for: Cholesterol Laundry of Cell Membrane and Fatty Liver by Detergent Liposomes to Improve Anti‐Cancer Drug Responsiveness of Patient Liver Tissues
Source: Adv Sci (Weinh). 2026 Jun 17;13(51):e76144. doi: 10.1002/advs.76144 (PMC13336731; doi:10.1002/advs.76144)
Supplement: Supplementary file 1 [file ADVS-13-e76144-s001.docx]

**Cholesterol laundry of cell membrane and fatty liver by detergent liposomes to improve anti-cancer drug responsiveness of patient liver tissues**

*Chansik Kim^†^, Joo Kyung Noh^†^, Sewoom Baek^†^, Seung Eun Yu, Jueun Kim, Seongyo Lee, Youngji Oh, Dai Hoon Han*, Seyong Chung*, Hak-Joon Sung**

**Supplementary figures**

**Supplementary figure 1.**

**Supplementary figure 2.**

**Supplementary figure 3.**

**Supplementary figure 4.**

**Supplementary figure 5.**

**Supplementary Figure 6**

**Supplementary Figure 7**

**Supplementary figure legends**

**Supplementary Figure 1**. **Determination of detergent (Triton X-100) concentration to add into the membrane of liposome (~100 nm in diameter).**

**(A)** As Triton 100-X concentration in the liposomal membrane increases beyond 0.13 mM, the circular morphology of the liposomes is disrupted under transmission electron microscopy (TEM). Scale bar = 100 nm.

**(B)** Cell viability also decreases below 80% when the Triton X-100 concentration exceeds 0.13 mM with validation of Triton X signals in the liposomal membrane. Together, 0.13 mM of Triton X-100 was selected for incorporation into the liposomal membrane.

**(C)** When 0.13 mM of Triton X-100 is incorporated into the membrane, the average size of detergent (+)-liposomes is 103 nm as determined by nanoparticle tracking analysis (NTA).

**Supplementary Figure 2. Characterization of cholesterol (+)-and (-)-liposomes.**

**(A)** Cholesterol (-)-liposomes exhibit circular morphology and 100 nm of diameter range under TEM, confirmed by NTA (avg. 98 nm). Scale bar = 100 nm.

**(B)** When membrane cholesterol levels are increased by culturing Hep3B in hypoxia, treatment of cholesterol (+)-liposomes significantly reduces the level compared to cholesterol (-)-liposome and No treat, whose levels are not significantly different from each other.

**(C)** After pre-treatment of liposomes, hydrophilic doxorubicin is treated to Hep3B cells by increasing the concentration for 24 h, and cell viability is assessed by CCK-8 assay. Only the highest concentration (10 μM) of doxorubicin significantly reduces cell viability by pre-treatment of cholesterol (-)-liposomes compared to No treat. In contrast, cholesterol (+)-liposomes support the anti-cancer effect of hydrophilic doxorubicin across 0.1, 1, to 10 μM as the cell viability significantly decreases compared to No treat. Data = mean ± SEM (N =3) Statistical significance is denoted by *p < 0.033, **p < 0.002, ***p < 0.001, and not significant (ns).

**(D)** Hemolysis of red blood cells (RBCs) is examined by treating cholesterol (+)-liposomes with incremental concentrations (0, 0.2, 0.4, and 0.8 mg/mL). As a reliability of the assay, the osmotic hemolysis of RBC is seen at 0.55% NaCl when the concentration is increased from 0 to 0.9%. Thus, when the hemolysis by 0.55% NaCl is set to 100%, the test concentration of cholesterol (+)-liposome in 0.55% NaCl exhibits no further hemolysis as the RBC viability is not significantly reduced compared to 0 mg/mL of cholesterol (+)-liposomes in 0.55% NaCl.

**(E)** These test concentrations of cholesterol (+)-liposome without NaCl exhibit non-significant cytotoxicity to human umbilical vein endothelial cells (HUVECs) as indicated by the viability maintenance compared to No treat. Data are presented as mean ± SD (n = 3). Statistical significance was assessed by Student's t-test (ns: not significant).

**Supplementary Figure 3.** **Hypoxic culture of Hep3B cells to accumulate lipid in the membrane**

**(A)** As hypoxia is intensified by depleting O_2_ in the culture of liver cancer cells, the membrane level of cholesterol increases.

**(B)** In alignment, when Hep3B cells are cultured in hypoxia (1% O₂) for 48 h, the cholesterol accumulation with Filipin staining significantly increases compared to normoxia under confocal imaging (left) with quantitative analysis (right). Scale bar = 50 μm. Data = mean ± SEM. N = the number of dots as independent replicates in each graph. Statistical significance is denoted by ***p < 0.001.

**Supplementary Figure 4.** **HCC patient tissues in 3D perfusion chips with hypoxic lipid accumulation.**

**(A)** When MRI-arterial phase imaging is carried out, O_2_ richness under arterial supply serves as a classification point for HCC patient livers within or above the normoxic range (CAP: controlled attenuation parameter). The CAP of the mid-hypoxic type (Patient #2) exceeds the normoxic range, indicating potential resistance to anti-cancer drugs and poor prognosis.

**(B)** The cancer statuses of hypoxic #3 and mid-hypoxic #2 livers are confirmed by the marker expression of CAIX which are significantly higher compared to the normoxic #1 one, as assessed by the images (top) and quantitative image analysis (down).

**(C)** Lipid accumulation with white vacuoles also increases significantly in hypoxic #3 and mid-hypoxic #2 livers compared to the normoxic #1 one, as assessed by H&E staining (top) with quantitative image analysis of the lipid droplet area (down). Scale bar = 200 μm Data = mean ± SEM. N = the number of dots as independent replicates in each graph. Statistical significance is denoted ***p < 0.001.

**Supplementary Figure 5.** **Superior liver accumulation of liposome by intravenous (IV) injection over intraperitoneal (IP) route in mice**

DiD-labeled liposomes are injected via two clinical routes for 24 h to compare liver targeting in C57BL/6 mice, so that fatty liver therapy can be supported. IV injection significantly increases liposome accumulation in the liver in addition to the spleen compared to IP, under IVIS after harvesting the organs (left) with quantitative analysis (right). Lung, kidney, and intestine exhibit no significant differences in liposomal accumulation between the two groups. Data = mean ± SEM (n =3). Statistical significance is denoted by *p < 0.033, **p < 0.002, and not significant (ns).

**Supplementary Figure 6. Validation of membrane cholesterol transfer to liposomes through fluorescence resonance energy transfer (FRET) with acceptor photobleaching.**

**(A)** FRET occurs when an excited donor fluorescent transfers the emission energy to an acceptor fluorescent within 10 nm distance under no acceptor bleaching, resulting in fluorescence emission of the acceptor as opposed to the donor (left). When the acceptor emission is photobleached, the energy transfer is stopped within 10 nm, resulting in an increase in the donor mission When the distance between the donor and acceptor increases over 10 nm, the emission of donor is not changed despite acceptor photobleaching. This strategy is approached (right) to validate the transfer of cholesterol (donor: green) from endosomal membrane to the liposome (acceptor: red). Acceptor photobleaching is expected to unchanged or increase the donor emission depending on no (over 10 nm) and the transfer (within 10 nm).

**(B)** Hep3B is cultured with endosomal BODIPY-cholesterol (green donor) and cholesterol (+)-liposomes with DiI membrane staining (red acceptor) and time-lapse imaged for 25 s under confocal microscopy. Acceptor photobleaching at 10 s in the yellow region of interest (ROI) validates the transfer as the donor green emission increases in contrast to the disappearance of acceptor red mission (white dotted lines: the cell boundary). Scale bar =10 μm.

**(C)** This result is confirmed through quantitative analyses of magnified ROI images before (top) and after (bottom) photobleaching. Scale bar = 2 μm.

**Supplementary Figure 7. siRNA knock-down of Rev-Erbα and CYP7A1 to validate their key roles in cholesterol clearance inside ER.**

**(A)** The knockdown efficiencies by transfecting siRNAs of NR1D1 (encoding Rev-Erbα) and CYP7A1 to Hep3B are confirmed as their mRNA expressions are significantly reduced compared to the corresponding scrambled siRNAs (control).

**(B)** The Rev-Erbα transcription factor mediates the CYP7A1 activity to clear cholesterols by converting to 7α-hydroxycholesterols for excretion from ER. Hence, their knockdowns using the siRNAs increase significantly cholesterol levels in ER compared to the corresponding scrambled siRNAs. Data = mean ± SEM (n=5). Statistical significance is denoted by **p < 0.002, ***p < 0.001, and not significant (ns).
